# A combined PIE-FRET and FCS assay to monitor RNA dynamics and cleavage by SARS-CoV-2 Nsp15

**DOI:** 10.21203/rs.3.rs-7717808/v1

**Published:** 2025-10-09

**Authors:** Kenya Gordon, Zoe Wright, Meredith Frazier, Benjamin S. Clark, Isha Wilson, Cameron R. Stephens, Irene Silvernail, Robin E. Stanley, Sharonda J. LeBlanc

**Affiliations:** 1 Department of Physics, North Carolina State University, Raleigh, NC 27695-8202, USA; 2 Molecular and Cellular Biology Laboratory, National Institute of Environmental Health Sciences, National Institutes of Health, Department of Health and Human Services, 111 T. W. Alexander Drive, Research Triangle Park, NC 27709, USA; 3 Department of Chemistry and Biochemistry, College of Charleston, 66 George St., Charleston, SC 29424, USA; 4 Current Affiliation: Howard University College of Medicine, Washington, DC 20059, USA

**Keywords:** FRET, FCS, Nsp15, RNA dynamics, single-molecule

## Abstract

Nsp15 is an endoribonuclease, highly conserved among coronaviruses, that helps the virus evade detection in host cells by cleaving uridine-rich viral RNA sequences. Those sequences would otherwise trigger immune response pathways. Its essential role in pathogenesis and highly conserved nature make Nsp15 an attractive target for therapeutic intervention. While its crystal structure and uridine specificity are well-established, the influence of RNA structural dynamics and divalent cations on Nsp15 activity remains less understood. Leveraging single-molecule Pulsed Interleaved Excitation (PIE)-FRET and Fluorescence Correlation Spectroscopy (FCS) in combination, we developed an assay to track RNA cleavage by Nsp15 variants in real time and monitor the conformational dynamics of hybrid RNA/DNA substrates. Using our methodology and analysis strategies, we obtained clear indicators RNA cleavage with both PIE-FRET and FCS data analysis. Our assay also revealed signatures of unique dynamic behavior in uridine-containing RNA substrates, indicating that divalent cations enhance substrate flexibility, which increases the reaction rate of RNA cleavage by Nsp15. We propose that divalent cations induce a more favorable conformation in single-stranded RNA substrates that mediate the efficient nuclease activity of Nsp15.

## INTRODUCTION

Several human coronaviruses (CoVs) cause serious human diseases including severe acute respiratory syndrome (SARS), Middle East respiratory syndrome (MERS), and most recently SARS-CoV-2 [1–3]. These CoVs are structurally composed of nucleocapsid proteins that assemble into a capsid structure encasing the viral genetic material with spike proteins protruding from a bilayer envelope that allow the viruses to recognize and invade target host cells [4]. As positive sense RNA viruses, CoVs contain single-stranded RNA that can be immediately translated when released into the host cell cytoplasm. The products of translation, polyproteins pp1a and pp1ab, then undergo proteolysis yielding 16 non-structural proteins (Nsps) that are responsible for a variety of functions including degrading and inhibiting translation of host RNA [5], generating new replication sites by modifying the endoplasmic reticulum (ER) of the host [6], and synthesizing more copies of viral RNA that are used to produce additional virions [7].

Nsp15, one of the better conserved nonstructural proteins in coronaviruses [8], is a homohexameric endoribonuclease that aids in the evasion of host immune systems by cleaving viral RNA during processing. The double-stranded RNA (dsRNA) intermediates produced during processing of the viral genome are known to elicit an immune response in host cells [9], but fragmentation of these viral RNA sequences by Nsp15 disrupts the immune response signaling pathways making viral replication more efficient [10,11]. Animal studies have shown that when CoVs are Nsp15-deficient, the immune response of the animal host is more effective against the viral infection, suggesting that Nsp15 plays an important role in supporting the proliferation of coronaviruses in host cells [12]. Thus, a better understanding of the enzymatic activity of Nsp15 may provide insight with applications for CoV therapies.

Nsp15 primarily cleaves 3’ of uridine (U) in RNA substrates, though the sites in coronaviral genomic and/or subgenomic RNA that are targeted by Nsp15 have been debated for over a decade [8,13,14]. Directly detecting cleavage products of Nsp15 during infection by coronaviruses that affect humans is prohibitively difficult, due to 1) the presence of multiple competing ribonucleases expressed by both the virus and host, and 2) the level of biohazard risk associated with culturing highly infectious pathogens. Characterization approaches that work around these challenges, such as studying Nsp15 homologs in non-human coronaviruses or recombinant Nsp15 overexpressed in non-pathogenic bacteria, have provided critically important windows into Nsp15’s mechanism of action, though each model system is necessarily limited in the information it can provide. Thus, multiple complementary experimental approaches are necessary to build a complete understanding of this protein.

Existing literature paints a picture of Nsp15 as a highly modular ribonuclease with a broad scope of possible substrates. In vivo models of infection (using cells infected with a mouse coronavirus, MHV) have detected Nsp15 cleavage sites in both the 5’ polyU tail of the negative strand [11] and regulatory regions of the positive strand of viral genomic RNA [15]. In vitro biochemical assays using purified recombinant SARS-CoV-2 Nsp15 have shown that Nsp15 can act on a variety of RNA substrates, including dinucleotides [16], short [17] and long single-stranded RNA (ssRNA) [18], and dsRNA [19]–though Nsp15 will cleave at different Us even in the same sequence of RNA, depending on whether that RNA is single- or double-stranded [19]. Structural studies using X-ray crystallography [20] and cryo-electron microscopy (cryo-EM) [21] have revealed that Nsp15’s active site is small and accommodates only the target U. Further, with the exception of the target U in the active site, Nsp15 forms only non-sequence specific interactions with dsRNA [14,19,22]. To bind dsRNA, Nsp15 uses modular H-bonding and electrostatic interactions with the RNA sugar phosphate backbone, and hydrophobic and aromatic interactions with the bases of the target U and its immediate neighbors. In fact, the binding efficiency of Nsp15 to dsRNA is not significantly affected by the absence of Us in the dsRNA [23], due to these non-sequence specific interaction modes. No structural data to date has captured interactions between Nsp15 and more than a few nucleotides of ssRNA [17,18,21], suggesting interactions with ssRNA are likely dynamic, variable, and/or transient. This body of literature has recently begun to converge on RNA structure and spontaneous RNA dynamics as critically important signals used by Nsp15 to select its cleavage targets [14,23]. For example, a recent study observed a correlation between spontaneous solvent accessibility of a U in dsRNA (assessed by NMR spectroscopy) and cleavage efficiency of that U by Nsp15 [14]. However, direct measurements of RNA dynamics in the absence and presence of Nsp15 have not been reported.

An additional ongoing question in the literature concerns the role of Mn^2+^ ions in Nsp15’s cleavage efficiency. While millimolar concentrations of Mn^2+^ increase the catalytic activity of Nsp15 [24], Mn-induced conformational changes of Nsp15 have not been observed in spectrographic assays [16]. Recent cryo-EM structures of Nsp15-substrate complexes also show no evidence of manganese within the Nsp15 active site [17,21], leaving the specific role of the manganese cofactor undetermined. The finding that the manganese co-factor is not implicated in direct interaction with Nsp15 [17,21] suggests that divalent ion interactions with the RNA substrate itself should not be overlooked. This is further supported by recent analyses that demonstrate Nsp15’s reliance on metal ions is substrate dependent, having a greater impact on ssRNA than dsRNA [18], and essentially no impact on dinucleotides [16]. Divalent metal cations are known to impact RNA structure and catalyze cleavage and binding reactions [25–27], and cations have been observed interacting with nucleotides both by binding site-specifically as well as contributing to atmospheric binding [28,29]. In addition, while interactions between metal ions and RNA nucleobases have been shown to stabilize RNA structure for cleavage by other RNA nucleases [30–32], the roles of ion environment in RNA substrate conformational dynamics and cleavage target recognition are not well studied for Nsp15.

To monitor the dynamics [33–35] of RNA substrates and its interactions with Nsp15, we employed simultaneous Fluorescence Correlation Spectroscopy (FCS) and Pulsed Interleaved Excitation Förster Resonance Energy Transfer (PIE-FRET). These experiments were informed by previous cryo-EM and biochemical studies [17,21], and provide an important complement to those techniques. FCS is a technique that utilizes intensity fluctuations of freely diffusing fluorescent reporters to experimentally characterize molecular dynamics such as diffusion [36–39]. Measuring FRET efficiency at the single molecule level allows for nanoscale spatial resolution and sub-millisecond time resolution of conformational changes and transient biological events [40,41]. PIE is a method by which two pulsed lasers excite the donor and acceptor of a FRET pair in an alternating pattern [42,43]. PIE-FRET allows for the detection of conformational states and dynamics of biomolecules diffusing in solution. Combined, the measurements provide the spatial and temporal resolution necessary to probe the transient dynamics of the RNA cleavage targets for Nsp15. Distinct from traditional biochemical assays, we can simultaneously track the subpopulations produced as cleavage of the RNA substrate occurs in the presence of Nsp15 variants, providing valuable insights into the role of substrate dynamics and divalent cations in the cleavage reaction. Interestingly, our biophysical approach enabled detection of uridine-dependent microsecond dynamics in an RNA substrate that are influenced by the presence of divalent cations and likely confer specificity of Nsp15 for cleaving uridine.

## MATERIALS AND METHODS

### Protein expression and purification

SARS-CoV-2 Nsp15 plasmids (WT, W333A, and H235A) were created as described in prior work [21]. Transformed C41(DE3) *Escherichia coli* (*E. coli*) competent cells were used to inoculate 10 mL of Terrific Broth (TB) containing 100 μg/mL ampicillin and grown at 37°C and 210 rpm to an optical density (OD) of 0.8. The starter culture was transferred to 1 L of TB containing 100 μg/mL ampicillin at 37°C and 210 rpm. At an OD of 0.2, the temperature was lowered to 16°C and after reaching target temperature, cells were induced with 0.5 mM isopropyl β-D-1-thiogalactopyranoside (IPTG). The cells expressed Nsp15 for ~16 hours overnight before harvesting. Cells were stored at −80°C until purification.

To begin purification, pelleted cells were resuspended in lysis buffer (50 mM Tris pH 8.0, 500 mM NaCl, 5% glycerol, 5 mM βME, 5 mM imidazole). A Roche cOmplete, EDTA-free protease inhibitor tablet (Sigma-Aldrich, St. Louis, MO, 11873580001) and 2 drops of Triton X were added to the cell slurry. Cell slurry was then sonicated for a total time of 6.5 minutes. After centrifugation for 50 minutes at 15000 rpm and 4°C, the cleared lysate was recovered. 2 mL of TALON metal affinity resin was equilibrated in lysis buffer and incubated with the cleared lysate for 45 min at 4°C. The solution was poured into a 10 mL gravity column and the beads were washed with lysis buffer. Elution buffer (50 mM Tris pH 8.0, 500 mM NaCl, 5% glycerol, 250 mM imidazole) was used to elute his-tagged Nsp15 from the TALON resin, and the elutions containing protein were combined and concentrated by centrifuging for 10 minutes at 3000 g. Nsp15 was then buffer exchanged with a PD-10 gravity column (Cytiva, Marlborough, MA, 17-0851-01) into a low salt buffer (50 mM Tris pH 8.0, 150 mM NaCl, 5% glycerol) prior to thrombin cleavage. Nsp15 in low salt buffer was supplemented with 2 mM βME, 2 mM CaCl_2_, and 20 units of thrombin (Sigma Aldrich, St. Louis, MO, T7513). The cleavage reaction was incubated for 4 hours on a shaker at room temperature. Afterwards, phenylmethylsulfonyl fluoride (PMSF) was added to quench the cleavage reaction, and the sample was passed through TALON resin after the beads were re-equilibrated in low salt buffer. A Superdex-200 column equilibrated in size exclusion chromatography (SEC) buffer (20 mM HEPES pH 7.5, 150 mM NaCl, 5 mM βME, 5 mM MnCl_2_) was used to isolate active Nsp15 hexamers. SDS-PAGE gels confirmed the presence of Nsp15 (monomer MW ~ 42 kDa).

### Nucleic acids

All oligos were purchased from Integrated DNA Technologies (IDT, Coralville, IA). We designed an RNA/DNA hybrid substrate labeled with a FRET pair (Atto550-Atto647N) to explore the cleavage activity of Nsp15 (Figure 1A). Both donor and acceptor are located on the ends of each oligo (substrate-substrate FRET). The RNA/DNA hybrid strand has 50 DNA bases and 10 RNA bases containing either an adenine (A) or uridine (U) base in the center (orange). The complementary DNA strand has 50 bases. The substrate was designed with a duplex DNA anchor and biotinylated end for future surface attached experiments. The oligo sequences are listed in Supplementary Table S1. The donor-labeled (Atto550) DNA strand (green) was annealed to the acceptor-labeled (Atto647N) hybrid DNA/RNA strand (green/pink) by heating equimolar amounts (10 μM) of each strand in annealing buffer (10 mM Tris pH 8, 50 mM NaCl, 1 mM EDTA) with a total volume of 10 μL to 95 °C for 5 minutes in a Bio-Rad T100 Thermal Cycler, followed by cooling to 25°C over 140 minutes at −1°C per 1-minute cycle, and then stored at 4 °C until further use. The Förster distance of the Atto550-Atto647N FRET pair was estimated to be 65 Å from FPbase assuming κ^2^ is 2/3 [44], and the expected separation between the donor and acceptor after annealing was approximately 37 Å, assuming a 0.34 nm separation per base. This donor-acceptor separation distance corresponds to a minimum FRET efficiency of approximately 0.97 for the high-FRET A and U substrates (HFA and HFU).

Mid-FRET substrates A and U (MFA and MFU) were designed to explore conformational dynamics of the substrates. Mid-FRET substrates consisted of an RNA/DNA hybrid end-labeled with Atto647N, and a complementary DNA strand containing an AF546-labeled thymine four bases from the 3’ end (Figure 1B, Supplementary Table S1). The use of AF546 in these constructs was based on availability of internal labels from the supplier. The hybrid strand contained 45 DNA bases and 15 RNA bases – including an A or U at the cleavage site located 12 bases from the 5’ end of the RNA/DNA hybrid. The mid-FRET substrates were annealed using the same protocol used for the high-FRET substrates. The Förster distance of the AlexaFluor546-Atto647N FRET pair was estimated to be 64.13 Å from FPbase assuming κ^2^ is 2/3. The separation between the donor and acceptor for mid-FRET substrates after annealing was approximately 60 Å, assuming a 0.34 nm separation per base. This corresponds to a minimum FRET efficiency of approximately 0.6.

Acceptor-labeled short oligos of 12 nucleotides (SO12) were purchased as a proxy for the cleaved substrate fragments of the high-FRET (HFU) substrate (Supplementary Table S1).

DNA oligomers with sequences from a benchmark single-molecule FRET study [45] were purchased from IDT with an internal amino modifier C6 deoxythymidine (dT) at the sites for fluorescent labeling (Supplementary Table S1). For labeling, we incubated each donor (D) or acceptor (A) oligomer with a 20x molar excess of the appropriate Atto-NHS ester dye (Sigma-Aldrich 92835 – Atto550, and 18373 – Atto647N) in a fresh sodium bicarbonate buffer in 18.2 MΩ double-deionized water, ddiH2O (adjusted to pH 8.5 with 6M HCl) and reacted overnight at 4°C. The oligomers were purified with a ZYMO DNA oligo purification kit (D4060), and the UV/Vis absorbance spectrum was measured to determine labeling efficiency. The labeled D and A oligomers were then annealed in equimolar amounts in 1x phosphate-buffered saline (PBS) by heating to 92°C for 4 minutes in a thermal cycler and cooling to room temperature at − 1°C per minute.

### Sample preparation and data acquisition

Nsp15 and fluorescently labeled RNA/DNA substrates were diluted to 5 nM in cleavage buffer (30 mM HEPES pH 7.5, 100 mM NaCl, 5 mM DTT, and 5 mM of divalent cation chloride solution). 40 mL droplets were then pipetted onto a coverslip, and to collect data the laser spot was focused 40 mm into the droplet. Acquisition times were 60–120 seconds, and samples were kept on ice between measurements that were taken at room temperature. For all experiments, the laser power density ranged from 2.4 – 12 kW/cm^2^.

### Time-resolved fluorescence microscopy and spectroscopy

Time-resolved fluorescence measurements were collected using a custom MicroTime 200 (PicoQuant, Berlin, Germany) with SymPhoTime64 software for data acquisition and analysis. The modular time-resolved confocal microscope system is built around an inverted Olympus IX-83 microscope body with a side port for optics and a piezoelectric z stage. It is equipped with three picosecond pulsed diode lasers (485, 531, and 636 nm) and a laser driver module (SEPIA II) capable of operating in pulsed (up to 80 MHz) and continuous wave (cw) modes. The lasers are cleaned up with narrowband filters, fiber-coupled into the main optical unit (MOU), directed to a main quad-band dichroic mirror, and focused onto the sample with a UPlanSApo – Superchromat 60× 1.2 numerical aperture (NA) water immersion lens (Olympus Corporation, Tokyo, Japan). A fast galvo beam scanning module (FLIMbee) with a 0.5 ms dwell time (lower limit) is used for laser beam scanning. Fluorescence from the sample is collected with the same objective and spatially filtered through an exchangeable circular confocal pinhole (100 mm used for these experiments) and directed to one or two single photon avalanche diodes (SPADs – Excelitas). For Förster/fluorescence resonance energy transfer (FRET) experiments with 531 and 636 nm laser excitation, the fluorescence is spectrally filtered using a dichroic mirror (635 long pass) placed between the two detectors and bandpass filters in front of each detector to separate donor (582/64, Channel 2) and acceptor (690/70, Channel 1) emission. For TCSPC, a multichannel event timer with 10 ps resolution and 650 ps deadtime (MultiHarp 150) in time-tagged time-resolved (TTTR) measurement mode applies time tags to individual photons detected at each SPAD, both relative to the laser pulse (nanotime) and relative to the start of the experiment (macrotime). Generating real-time histograms of the different time tags enables simultaneous fluorescence intensity, lifetime, and correlation data collection.

Operating the lasers in pulsed interleaved excitation (PIE) mode enables selective excitation and detection of separate donor, acceptor, and FRET signals on the nanosecond (ns) time scale. The laser excitation is synchronized in the SymPhoTime software such that the 531 and 636 nm lasers pulse in an alternating manner, successively exciting acceptor and donor molecules directly. Detected single photons are associated with the exciting laser pulse and the arrival detector to separate donor, acceptor, and FRET signals.

### Fluorescence Correlation Spectroscopy (FCS)

Emitted photons from donor and acceptor reporter fluorophores were separated into different detection channels (Channel 1 and Channel 2), binned into 1 millisecond time bins, and the donor signal after direct excitation ($I_{GG}$), acceptor signal after direct excitation ($I_{RR}$), acceptor signal after donor excitation (FRET, $I_{GR}$) was autocorrelated using Eq. 1:

(1) $$G(\tau )=\frac{\left\langle F(t)F(t+\tau )\right\rangle }{{\left\langle F(t)\right\rangle }^{2}}-1$$

Where <> represents the time average, τ is the lag time, F is the fluorescence intensity, and G(τ) is a measure of the self-similarity of the signal [37,46].

Intensity autocorrelation curves were fit using PIE Analysis with MATLAB (PAM) [47], Python, and/or SymPhoTime 64 software using either Eq. 2, a pure diffusion fitting model [48], or Eq. 3, a conformational fitting model [49]:

(2) $$G(\tau {)}_{\mathrm{pure}\;\mathrm{diffusion}}=\frac{1}{N}\cdot \frac{1}{1+\frac{\tau }{\tau_{D1}}}\cdot {\left(\frac{1}{1+\frac{\tau }{\tau_{D1}}\cdot \frac{1}{\kappa^{2}}}\right)}^{\frac{1}{2}}$$

(3) $$G(\tau {)}_{\mathrm{conformational}}=\frac{1}{N}\cdot \frac{1+A_{c}\left(e^{-{\left(\frac{\tau }{\tau_{c}}\right)}^{\beta_{c}}}\right)}{1+\frac{\tau }{\tau_{D2}}}\cdot {\left(\frac{1}{1+\frac{\tau }{\tau_{D2}}\frac{1}{\kappa^{2}}}\right)}^{\frac{1}{2}}$$

where τ is the correlation time, $\tau_{D1}$ and $\tau_{D2}$ are diffusion times, N is the number of molecules, $\beta_{C}$ is the stretch factor, $\tau_{C}$ is the relaxation time of conformational dynamics, and $A_{C}$ is related to the equilibrium constant for the conformational change. To convert diffusion time to diffusion coefficient, D, the confocal volume aspect ratio, characterized by κ, was determined for the 531 and 636 nm lasers via an FCS calibration in SymPhoTime 64 for measurements collected from a solution of either Atto550 or Atto655-maleimide with known diffusion coefficients of 375 mm^2^/s and 407 mm^2^/s, respectively [50] (Supplementary Table S2).

Average diffusion times were calculated for HFU and HFA (Eq. 1 and Eq. 3), short oligos (SO) representing RNA cleavage fragments (Eq. 1 and Eq. 2), and the acceptor-labeled single-strand RNA/DNA hybrids (Eq. 1 and Eq. 3) with SymPhoTime 64 to assess the sensitivity of FCS-determined diffusion times to biomolecule size (Supplementary Figure S1). HFU fluorescence intensity data collected upon initial incubation with Nsp15 and after 2 hours of incubation were autocorrelated and fit to Eq. 3 in each analysis software to confirm agreement between diffusion parameters calculated in each software (Supplementary Table S3). To monitor cleavage of HFU and HFA by different mutants of Nsp15 over time, the fluorescence autocorrelation data of the substrates were fit to a mixed model consisting of a combination of Eq. 2 and Eq. 3 using Eq. 4:

(4) $$G(\tau {)}_{\mathrm{mixed}}=A\;*\;G(\tau {)}_{\mathrm{conformational}}+(1-A)\;*\;G(\tau {)}_{\mathrm{pure}\;\mathrm{diffusion}}$$

where A is equal to the square of the fractional intensity of species 1 (${f_{1}}^{2}$, un-cleaved substrate)[51]. We wrote a custom Python script to perform the mixed-model fitting utilizing a curve fitting method of least squares regression. The characteristic diffusion time of cleaved substrates was estimated by measuring the short oligo (SO12) sample and fitting the autocorrelated data to Eq 2. The extracted fit parameters (Supplementary Figure S1B) were used in the pure diffusion component of the mixed model. The characteristic fitting parameters of the conformational component of the mixed model, $\tau_{D2},\;\beta_{C},\;\tau_{C}$, and $A_{C}$ were calculated independently for each combination of high-FRET substrate and Nsp15 variant. At the beginning of each experiment, we collected three 90-second FCS measurements. This initial triplicate data was independently autocorrelated and fit to Eq. 3 (acceptor signal only, $I_{RR}$, to monitor the RNA). The average of each fit parameter was used in the conformational component of the mixed model (Supplementary Table S4). The parameter of interest from the mixed model utilized to monitor cleavage over time was A in Eq. 4. The mixed model approach was also used for FCS analysis of HFU substrates incubated with wildtype (WT) Nsp15 under different divalent ion conditions.

### Pulsed Interleaved Excitation Förster Resonance Energy Transfer (PIE-FRET)

FRET relies on the distance-dependent intensity or fluorescence lifetime changes of fluorescent reporters due to non-radiative energy transfer to report on the spatial separation between the fluorescent molecules [52]. The efficiency of that energy transfer depends on the fluorophore separation distance, d, as $E_{\mathrm{FRET}}={\left[1+{\left(d/R_{0}\right)}^{6}\right]}^{-1}$, where $R_{0}$ is the Förster radius, the distance at which a specific FRET pair achieves 50% FRET efficiency [53]. In pulsed interleaved excitation (PIE), picosecond pulsed diode lasers alternate pulses on the nanosecond timescale. Photons are collected for each excitation window allowing data to be recorded for both donor and acceptor excitation wavelengths quasi-simultaneously. The sample fluorescence is allowed to decay completely before excitation by the next laser pulse to minimize crosstalk. Using PIE, low-FRET populations can be distinguished from donor-only populations by filtering for acceptor photons that are collected during donor excitation [54], and more complex samples containing mixed populations or dynamic FRET states can be monitored more effectively.

PIE-FRET data collection and analysis suppress the contribution of donor-only and acceptor-only populations with proper determination of the leakage,a, and direct excitation, d, factors for the Atto550-Atto647N or AF546-Atto647N FRET pairs. The leakage factor, a, and direct excitation factor, d, for corrected PIE-FRET analysis were determined for AF546, Atto550, and Atto647N as described previously [45] (Supplementary Table S2). The detection efficiency, γ, and excitation correction factor, β, for PIE-FRET analysis were determined independently (Supplementary Figure S2) for individual substrate conditions by fitting inverse stoichiometry versus FRET efficiency data for a detection efficiency value of 1 to Eq. 5 [55]:

(5) $$\frac{1}{S}=\Omega +\Sigma \cdot E$$

S is the raw stoichiometry of photon bursts, where the raw stoichiometry describes the ratio of photons emitted after donor excitation to the sum of photons emitted after donor excitation and photons emitted after acceptor excitation. E is the FRET efficiency, and Ω and Σ are linear fit parameters. The fit parameters are related to the detection efficiency and excitation correction factor by the following Eq. 6 and Eq. 7 [55]:

(6) $$\gamma =\frac{\Omega \;-\;1}{\Omega \;+\;\Sigma \;-\;1}$$

(7) β=Ω+Σ−1

The inverse stoichiometry versus efficiency plots and corresponding correction factors for all experimental conditions are shown in Supplementary Figure S2. The corrected stoichiometry is given by the following equation once correction factors are determined:

(8) $$S=\frac{\gamma \;\times \;I_{GG}\;+\;I_{GR}\;-\;\alpha \;\times \;I_{GG}\;-\;\delta \;\times \;I_{RR}}{\gamma \;\times \;I_{GG}\;+\;I_{GR}\;-\;\alpha \;\times \;I_{GG}\;-\;\delta \;\times \;I_{RR}\;+\;\beta \;\times \;I_{RR}}$$

where $I_{GG},\;I_{GR}$, and $I_{RR}$ are photon burst counts of the donor after donor excitation, the acceptor after donor excitation, and the acceptor after acceptor excitation, respectively.

To capture the FRET efficiency states present in samples, PAM was used to employ an All Photon Burst Search (APBS) algorithm [56] utilizing 250 μs binning for imported time trace data collected in SymPhoTime 64. The background scattering and IRF measurements, used for fitting lifetime data, were determined from measurements focused within plain buffer droplets and on glass slides, respectively. The time-correlated single photon counting (TCSPC) histograms determined from the background corrected photon burst searches were fit for acceptor and donor photons separately to obtain fluorescence lifetime information. This data, in conjunction with the estimated Förster radii (determined from FPbase), correction factors, and fluorescence lifetimes (Supplementary Table S2) were used in PAM to determine Stoichiometry vs Efficiency histograms. Gaussian fitting of the FRET efficiency distributions was also performed in PAM using a maximum likelihood estimation (MLE) algorithm. For the FRET efficiency versus donor lifetime plots created in PAM, the static FRET line used as a reference for dynamic behavior are produced from Eq. 9 [35,57–59]:

(9) $$E=1-\frac{{\left\langle \tau_{D(A)}\right\rangle }_{F}}{\tau_{D(0)}}$$

where E is the FRET efficiency, ${\left\langle \tau_{D(A)}\right\rangle }_{F}$ is the intensity-weighted average lifetime of the donor in the presence of the acceptor, and $\tau_{D(0)}$ is the lifetime of the donor-only labeled oligo alone. The FRET efficiencies for the stoichiometry versus FRET efficiency histograms were produced from Eq. 10:

(10) $$E=\frac{I_{GR}\;-\;\alpha \;\times \;I_{GG}\;-\;\delta \;\times \;I_{RR}}{\gamma \;\times \;I_{GG}\;+\;I_{GR}\;-\;\alpha \;\times \;I_{GG}\;-\;\delta \;\times \;I_{RR}}$$

where α and δ are leakage and direct excitation correction factors, respectively (Supplementary Table S2)[45].

## RESULTS

### PIE-FRET and FCS report on cleavage and conformational dynamics of U-containing substrates

In this work, we used diffusion-based single-molecule spectroscopy to monitor the cleavage of RNA substrates by SARS-CoV-2 Nsp15, towards a more in-depth understanding of the cleavage mechanism. We developed a versatile fluorescence-based assay by designing donor-acceptor labeled RNA/DNA hybrid substrates (HFA, HFU, MFA, MFU, Figure 1 and Supplementary Table S1) to investigate the impact of Nsp15 mutation on cleavage efficiency, and the influence of divalent cation environment on the reaction. The ability to measure PIE-FRET and FCS on these substrates simultaneously enables reporting cleavage by observing a shift in PIE-FRET efficiency, and real time tracking of reaction subpopulations based on size (Figure 1D) by isolating and autocorrelating the donor, acceptor, or FRET signals separately. In addition, with time-resolved analysis of the PIE-FRET data, we detected RNA substrate dynamics that are likely key for uridine discrimination and cleavage.

**Figure 2.**
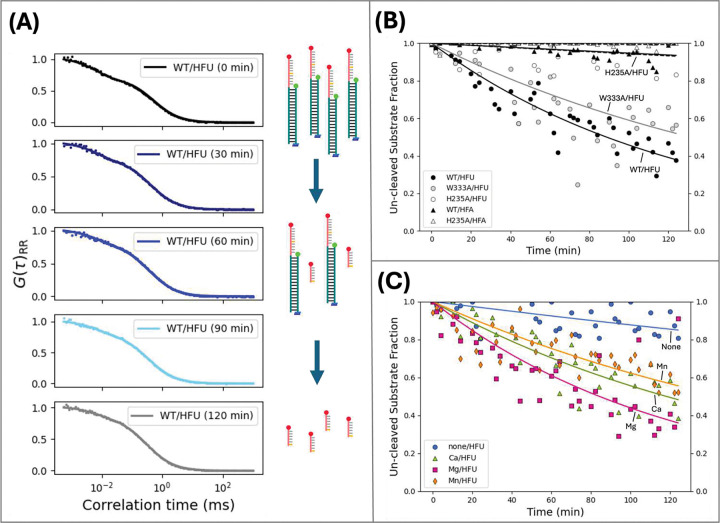
A) Example mixed model fits with Eq. 4 for WT/HFU at selected timepoints. Mixed model fitting parameters for all conditions and timepoints are included in Supplementary Table S7. B) Substrates HFA (triangles), and HFU (circles) were incubated with a 1:1 concentration of WT (black), W333A (gray), or H235A (white) Nsp15. An FCS curve fitting model with pure diffusion and conformational components, Eq. 4, was used to fit the autocorrelated acceptor signal ($I_{RR}$). The acceptor signal after direct acceptor excitation tracks the cleaved RNA-Atto647N fragment (Figure 1). The fraction of un-cleaved substrate determined by the mixed-model fit and plotted over 2 hours for each substrate and Nsp15 variant condition. Our analysis shows that the H235A mutant did not cleave HFA or HFU substrates, as expected. The fraction of un-cleaved HFU substrate decreased from 1 to approximately 0.4 when incubated with WT Nsp15 for 2 hours. The W333A mutant cleaved less than half of HFU when incubated for equal time. Over the 2-hour time periods, three measurements were taken every 10 minutes. Each point in the plot represents the un-cleaved fraction, A, calculated with Eq. 4 for one 90 – 120s measurement and fit. The curves are exponential fits to the data (exponential fit parameters in Supplementary Table S8). C) HFU substrates incubated with wildtype Nsp15 in solution with 0 mM divalent cation (blue circles), 5 mM Ca^2+^ (green triangles), 5 mM Mg^2+^ (pink squares), 5 mM Mn^2+^ (orange diamonds). WT Nsp15 cleaved HFU under all divalent ion conditions, though none/HFU was cleaved least efficiently. Each point in the plot represents the un-cleaved fraction, A, calculated with Eq. 4 for one 90 – 120s measurement and fit. Mixed model fitting parameters are included in Supplementary Table S9. The curves are exponential fits to the data (fit parameters in Supplementary Table S10). Data was not collected for none/HFU between 30 and 45 minutes.

In all experiments, the hybrid RNA/DNA oligo has an acceptor on the 5’ RNA end, and cleaved RNA is tracked by isolating the acceptor fluorescence signal upon direct excitation - $I_{RR}$. The complementary DNA oligo facilitates addition of a donor molecule and a biotin site for future surface attached studies. Upon annealing, the 10-base separation between the carefully selected donor-acceptor FRET pair (Atto550-Atto647N) leads to a high FRET signal (E~1, Figure 2A) for HFA/HFU. The 18-base separation between the donor-acceptor FRET pair (AF546-Atto647N) for MFA/MFU substrates was designed to be sensitive to conformational changes in the RNA, leading to a mid-FRET signal (0.4<E<0.8). Importantly, these experiments were conducted on diffusing molecules, making sample preparation straightforward.

To explore nucleotide base specificity of Nsp15, high-FRET substrates containing either an adenine (HFA) or uridine (HFU) in the RNA sequence (Figure 1A, B, Supplementary Table S1) were incubated with equimolar wildtype Nsp15 and monitored while diffusing in solution for 2 hours. Previous work showed that Nsp15 has a strong specificity for cleaving RNA 3’ of uridines [11,17]. Substrates were diluted to 5 nM and PIE-FRET and FCS were collected simultaneously on a droplet of diffusing molecules (Figure 1C) to report cleavage by Nsp15. First, 40 mL of the substrate-protein solution was pipetted onto a thin coverslip and excited via PIE at 20 MHz, and three consecutive 90-second measurements were collected. Triplicate 90-second measurements were then taken every 10 minutes for 2 hours. Upon cleavage by Nsp15, separation of the donor and acceptor should result in the emergence of donor-only and acceptor-only populations in two-dimensional (2D) FRET efficiency (E, Eq. 10) - photon stoichiometry (S, Eq. 8) histograms [45]. We will refer to those plots as 2D E−S histograms. For acceptor-only labeled molecules S=0, for donor-only labeled molecules S=1, and for donor-acceptor pairs S=0.5. In practice, 0.3<S<0.8 can be attributed to single donor-acceptor labeled species[60]. Over the course of the 2-hour reaction, multiple species may be present in the solution including unannealed substate, annealed substate, cleaved substrate, Nsp15-RNA bound complexes, and the cleaved RNA product (Figure 1D). Using the combination of PIE-FRET analysis and FCS fitting to a mixed model, Eq. 4, we can track all labeled species over the course of the experiment.

PIE-FRET efficiency histograms of diffusing RNA substrates report cleavage by Nsp15 as a binary assay (high FRET = un-cleaved and no FRET = cleaved). Figure 2A shows the PIE-FRET efficiency histograms for the annealed intact and cleaved HFA and HFU substrates. Each 2D E−S histogram is the result of thousands of fluorescently labeled RNA/DNA molecules diffusing through the confocal volume over the course of the acquisition. As expected for the high-FRET substrates, the FRET efficiency histogram for annealed HFA and HFU substates is initially peaked near E=1, consistent with the 10-base separation between fluorescent reporters. After 2 hours of incubation with WT Nsp15, the 2D E−S histogram for HFA remains unchanged while that of HFU shows donor-only and acceptor-only subpopulations emerge at S>0.8, and S<0.2, respectively. The decreased subpopulation at E~1,S=0.5 for HFU is due to spatial separation of the FRET pair and indicates cleavage of the RNA. Fluorescence lifetime analysis also indicates cleavage by monitoring the change in the donor fluorescence lifetime of the entire ensemble over the course of an acquisition (Supplementary Figure S3). HFA remains un-cleaved, consistent with the preference of Nsp15 for uridine as a cleavage site as observed previously [17]. It is challenging to monitor the reaction over time with a binary FRET readout; thus, we applied fluorescence correlation spectroscopy (FCS) analysis to the same data set to track the evolution of reaction products.

Along with PIE-FRET data that reports cleavage, we obtain simultaneous FCS data, which we analyzed to track cleavage and subpopulations present in solution over the course of the experiment based on their physical size (Figure 1D). FCS measures fluorescence intensity fluctuations of reporter molecules as they diffuse through the confocal observation volume. The characteristic time scales over which these fluctuations occur correspond to specific types of dynamic behavior including diffusion at millisecond timescales, conformational dynamics at microsecond timescales, and rotational dynamics at nanosecond timescales [61]. FCS analysis can be used to extract specific parameters related to biomolecules of interest including concentration, diffusion time ($t_{D}$), and interconversion time between states by means of theoretical fitting models [46,62]. We have used the translational diffusion time ($t_{D}$) and the conformational relaxation time ($t_{C}$) extracted from fitting to characterize our substrates in this work. The translational diffusion time occurs on the millisecond timescale and indicates the average time a molecule dwells in the confocal volume before diffusing away. Smaller molecules have a short diffusion time, while larger molecules have a longer diffusion time. The conformational relaxation time is described later.

The clear disparity in size of the un-cleaved and cleaved HFU substrates enables monitoring cleavage products over the course of the reaction with FCS analysis. For this analysis, we focused on the signal from the RNA-Atto647N acceptor ($I_{RR}$) to report cleavage, which is initially attached to the RNA/DNA hybrid strand. This enables tracking the RNA-Atto647N fragment even after the FRET signal ($I_{GR}$) for an individual molecule disappears due to cleavage by Nsp15 (Figure 2A). For FCS analysis, the fluorescence intensity traces are autocorrelated using Eq. 1, then fit to an appropriate diffusion model, Eq. 2 or Eq. 3. The FCS fits obtained for Nsp15 variants incubated with HFU are shown in Figure 2B. For clarity, only the model fits for $I_{RR}$ are shown in the main text, but the autocorrelated data is also shown in Supplementary Figure S4 with fit parameters in Table S5. The autocorrelation curves with fits for the additional $I_{GR}$ and $I_{GG}$ signals are shown in Supplementary Figure S4, with fit parameters in Supplementary Table S5. The qualitative differences in the FCS curves of HFU alone pre-cleavage (Figure 2B, solid black) and short oligo 12 (SO12) ‘post-cleavage’ (Figure 2B, solid orange) are visibly apparent. SO12 is a Atto647N-labeled 12-nucleotide oligo which serves as a proxy for the completely cleaved substrate fragment of the HFU substrate. A decay in the autocorrelation curve characterizes dynamics in the fluorescently labeled species across different timescales [46]. To highlight differences, we normalized the FCS curves in Figure 2B to $G\left(2\cdot \tau_{D}\right)$, a lag time where the contribution from fast dynamics has effectively decayed and the curve is dominated by slower molecular diffusion [63,64]. We observe that the curve for HFU alone (Figure 2B, black solid) decays at both microsecond and millisecond timescales, thus a pure diffusion model is insufficient to fit the data. In contrast, the SO12 curve (Figure 2B, orange solid) only decays at the millisecond timescale and fits to a pure diffusion model, Eq. 2, yielding a diffusion time of 0.23 ms.

Microsecond timescale features in FCS curves can be attributed to photophysical effects such as photoblinking and triplet state occupancy [65], or conformational dynamics [37,61]. To explore the origin of the microsecond timescale features in FCS curves observed for HFU experiments (Figure 2B), we performed several control experiments. We constructed an acceptor-only substrate by annealing the hybrid high-FRET RNA/DNA strand with an unlabeled DNA complement (No-FRET A/U, NFA/NFU, Table S1). In the presence of divalent cations (Ca^2+^, Mg^2+^, and Mn^2+^), Trolox, and glucose oxidase/catalase (GOX/CAT), the microsecond timescale feature is not present for the NFA/NFU constructs (Supplementary Figure S5 and Table S6). We concluded from those experiments that the microsecond timescale feature in the autocorrelated $I_{RR}$ signal is only present when the donor is on the complementary strand and remains when a triplet state quencher or oxygen scavenging system is used. We therefore ruled out photoblinking and triplet state occupancy as the origin of the microsecond timescale feature and attributed it to RNA substrate conformational dynamics.

Although intensity fluctuations in the $I_{RR}$ signal are unexpected, these findings suggest that the fluctuations are likely due to a proximity-dependent photophysical interaction with the donor molecule which results from substrate dynamics. We note that the microsecond timescale feature is also present in the $I_{GR}$ signals pre-complete cleavage (Supplementary Figure S4). The photophysical origin of the detected fluorescence fluctuations in $I_{RR}$ in the presence of the donor molecule will be the subject of a separate manuscript. We therefore applied a conformational fit (Eq. 3) to the FCS curves of HFU substrates shown in Figure 2B (except for the SO12 control). The diffusion time for HFU alone (without Nsp15 added) determined from the conformational fitting model, Eq. 3, was ~ 0.6 ms, compared to 0.23 ms for SO12. This difference in diffusion time is due to the significant dissimilarity in size (Supplementary Table S1) between the cleaved fragment, represented by the SO12 substrate, and the annealed RNA/DNA molecules. The larger annealed HFU dwells longer in the confocal volume. Collectively, our findings suggest that the molecules undergo conformational changes while in the confocal volume, and the $t_{C}$ fit parameter from a conformational fit should capture the relaxation lifetime of transitions between states. Across experiments, $t_{C}$ ranges from 5 – 14 μs (Table S5). We expect that as more HFU molecules are cleaved over the course of the 2-hour reaction, the microsecond timescale conformational dynamics apparent in the FCS curves for the HFU reaction (Figure 2B, S4) will become less pronounced since the cleaved RNA fragment (represented by SO12) is a short sequence.

We analyzed FCS data for HFU substrates in the presence of Nsp15 variants. Autocorrelation curves for the acceptor fluorescence intensity ($I_{RR}$) were calculated for HFU incubated with wildtype, W333A, or H235A Nsp15. Both W333 and H235 lie in the active site of Nsp15, and prior work confirming the importance of these residues for efficient cleavage of RNA [17,19,21] motivated the selection of these mutants for further exploration. We measured HFU alone and a short control oligo (SO12, Supplementary Table S1) as a proxy for cleaved HFU (Supplementary Figure S4) as described above. Figure 2B shows representative FCS fits for HFU alone, SO12, HFU at the onset of incubation with Nsp15, and HFU after 2 hours of incubation with Nsp15 for wildtype, W333A, and H235A mutants. The autocorrelation curves and fits are shown in Supplementary Figure S4 and the fit parameters are listed in Supplementary Table S5. The parameters determined from the conformational fits were: translational diffusion time ($t_{D}$), conformational relaxation time ($t_{C}$), and the $A_{c}$ parameter, related to the relative quantum yields of the dye in both conformational states and the equilibrium constant for the state change.

A comparison of the 0-hour and 2-hour FCS fits for WT, W333A, and H235A Nsp15 mutants confirms that the autocorrelation curve shape and acceptor diffusion time changes when cleavage by Nsp15 occurs. The short control oligo (SO12) autocorrelation curve was fit to a pure diffusion model, Eq. 2, since the curve appeared to decay at the millisecond timescale only. All other autocorrelation curves (except the SO12 control data) fit to a conformational model, Eq. 3, due to a notable additional decay in the autocorrelation at microsecond timescales, suggesting conformational dynamics of the substrate as discussed above [61]. The FCS fit of HFU incubated with WT Nsp15 for 2 hours (Figure 2B, brown dashed) clearly shows a change in curve shape compared to the initial case (Figure 2B, Figure S4, brown solid), and a decrease in diffusion time from approximately 0.5 to 0.31 ms (Supplementary Table S5), suggesting near-complete substrate cleavage. The H235A mutant has an active site mutation that abolishes Nsp15 function as shown in previous work [17], therefore we do not expect cleavage of the HFU substrate. Consistent with that, no change in shape of the autocorrelation curve was observed for H235A (Figure 2B, Figure S4, red), and the diffusion times were determined to be approximately 0.56 ms for initial and final measurements (Supplementary Table S5). Fitting the autocorrelation curve for HFU incubated with W333A gave diffusion times of 0.45 and 0.38 ms for initial and final measurements respectively, but there was no notable reduction in the appearance of microsecond timescale decay (Figure 2B, blue), suggesting incomplete cleavage.

We propose that the conformational dynamics of the RNA substrate prior to cleavage are important for specific recognition at uridine and cleavage by Nsp15. Later, we show with time-resolved PIE-FRET analysis and analysis of the FCS $t_{C}$ parameter distinct signatures of dynamic behavior that are RNA sequence and divalent cation dependent. The final diffusion times for HFU incubated with WT Nsp15 compared to W333A were not significantly different, so we further investigated the impact of these mutations on Nsp15 cleavage efficiency by analyzing the kinetic evolution of subpopulations over the 2-hour reaction period with a mixed component diffusion model, Eq. 4.

### Nsp15 mutants and divalent cation environment impact Nsp15 cleavage efficiency

The endpoint diffusion times determined for HFU incubated with wildtype Nsp15 and W333A (Figure 2B, Table S5) cannot characterize reaction kinetics. To distinguish between the cleavage efficiency of Nsp15 variants, we developed a method to separate the contributions from different substrate subpopulations (Figure 1D) to the FCS autocorrelation curves. Our method of identifying and tracking cleavage reaction species by their diffusion time ($t_{D}$), and thus their size is a unique approach to kinetic analysis with PIE-FRET/FCS. We wrote a custom Python script to fit the autocorrelation curves produced from the acceptor signal after direct acceptor excitation $\left(I_{RR}\right)$ to a mixed FCS diffusion model, Eq. 4. We focus on the $I_{RR}$ signal in this analysis since it enables us to track the RNA-Atto647N molecules before and after cleavage over the course of the reaction. By separating the contributions into pure diffusion (cleaved) and conformational (un-cleaved) with the mixed model, we estimated the percentage of un-cleaved substrate in the reaction over time (Figure 3).

To explore the cleavage of the different mutants over time, we analyzed each of the triplicate 90-second measurements taken at 10-minute increments over 2 hours for HFA and HFU. The HFU and HFA substrates were incubated with either WT, W333A, or H235A Nsp15. The autocorrelation curves calculated from each FCS measurement were then fit to the mixed pure diffusion and conformational model, Eq. 4, to separate multiple subpopulation contributions and track the relative populations of cleaved and un-cleaved RNA over time. The average diffusion times of the un-cleaved HFA and HFU substrates and the cleaved portion (represented by SO12) were calculated from control experiments (Table S5). The relative fractions of each population were then determined from the mixed model fit using Eq. 4. Representative autocorrelation curves and mixed model fits for WT Nsp15 incubated with HFU at 0, 30, 60, 90, and 120 minutes are shown in Figure 3A. The weighting factors, A and 1−A in Eq. 4 are attributed to the conformational (un-cleaved) and pure diffusion (cleaved) contributions to the diffusion model, respectively.

The mixed model fitting untangled the relative differences in cleavage efficiency for WT, W333A, and H235A Nsp15 incubated with HFU. Figure 3B shows the un-cleaved substrate fraction, A, plotted as a function of time for HFA and HFU substrates incubated with WT, W333A, and H235A Nsp15 variants. The fitting parameters for the mixed model determined for each time point can be found in Supplementary Table S7. The diffusion time of the cleaved RNA was estimated by measuring the short control oligo (SO12) with the same sequence from the RNA/DNA hybrid as described above (0.23 ms). As Figure 3B shows, the fraction of un-cleaved HFU decreases faster over time for HFU incubated with wildtype Nsp15 (black closed circles) compared to W333A (gray closed circles). After 2 hours of incubation, the WT/HFU sample is approximately 35% un-cleaved compared to 70% un-cleaved for the W333A/HFU sample. With the mixed model analysis of FCS data, while W333A and WT Nsp15 are both active, the un-cleaved fraction of HFU incubated with WT decreased faster than W333A/HFU, indicating more efficient cleavage, consistent with biochemical experiments [17]. In addition, the percent un-cleaved at the endpoint was higher for W333A/HFU, suggesting that the cleavage reaction was incomplete at the 2-hour timepoint. It was previously determined that the W333 residue of Nsp15 engages with the post cleaved uridines of ssRNA substrates [17] and helps to stabilize the flipped uridine of dsRNA to support cleavage [19], suggesting that the W333A mutation may negatively impact cleavage efficiency. The fraction of un-cleaved HFU showed no change when incubated with H235A (Figure 3B, open circles). In prior work, the H235 residue, present within the active site of Nsp15, has been identified as a part of the catalytic triad necessary for nucleotide binding, and the H235A mutation was shown to inactivate Nsp15 cleavage capability [17].

The exponential fit parameters for all experiments in Figure 3B are given in Supplementary Table S8. In recent work, the kinetic rate for Nsp15 cleavage of a ssRNA substrate was reported to be about 0.05 min^*−1*^ for a 10:1 ratio of substrate to Nsp15 without manganese [16]. The cleavage rate determined from the exponential fit of WT/HFU (Figure 3B) in our work was approximately 0.009 min^*−1*^. Considering that our substrate to Nsp15 ratio is 1:1, that result is in good agreement with the previous biochemical study. This highlights the sensitivity of our method that uses small amounts of nucleic acid substrate and protein. We have shown that our mixed FCS model analysis approach can tease out different activity of Nsp15 mutants and determine kinetic rates, which can be applied to study other perturbations.

We applied our FCS mixed model fitting approach to explore the role of different divalent cations on Nsp15 cleavage activity. Mn^2+^ is well known as the preferential cofactor for Nsp15 homologs [16,24,66], but prior studies have shown that manganese neither induces conformational changes in Nsp15 [16] nor is implicated at the binding site during RNA cleavage [17]. We used the HFU substrate and collected simultaneous PIE-FRET and FCS as described above, then applied the mixed model fitting, Eq. 4. The results are consistent with a prior study finding that the cleavage product accumulates more quickly in the presence of divalent ions calcium, magnesium, and manganese compared to the substrate in solution without divalent ions [67]. Figure 3C shows the un-cleaved fraction, f, of HFU incubated with and without divalent cations over the course of 2 hours. Figure 3C shows that over the course of 2 hours, the fraction of un-cleaved HFU in divalent cation-free buffer (none/HFU) decreased to approximately 0.7. After 2 hours of incubation with WT Nsp15, the un-cleaved substrate fractions for Ca^2+^/HFU, Mg^2+^/HFU, and Mn^2+^/HFU were determined to be between 0.35 and 0.45. The mixed model fitting and the exponential fit parameters for Figure 3C are provided in Supplementary Tables S9 and S10, respectively. The cleavage rates in the presence of divalent cations are faster compared to no divalent cations, but we could not distinguish them from one another in that experiment. In complementary experiments on mid-FRET substrates (MFA/MFU), we discovered signatures of dynamic substrate behavior heavily influenced by the presence of divalent cations.

### Divalent cations contribute to substrate dynamics

The prospect of dynamic behavior in the intact HFA/HFU substrates revealed in FCS curves (Figure 2B) led us to explore the dynamics of the substrate in further detail. The presence of a decay feature at the millisecond timescale in the autocorrelation curves suggested conformational changes. While traditional biochemical assays can report on substrate cleavage, the role of RNA substrate dynamics and divalent cations is harder to investigate with traditional methods. We designed substrates with mid-FRET (MFA and MFU) for dynamic experiments. In addition, since the role of manganese in facilitating Nsp15 recognition and specific cleavage at uridine is not well understood and manganese is not known to bind to Nsp15 [17,21,66], we explored the impact of the divalent cation environment on the conformational dynamics of the substrates in the absence of Nsp15.

**Figure 4.**
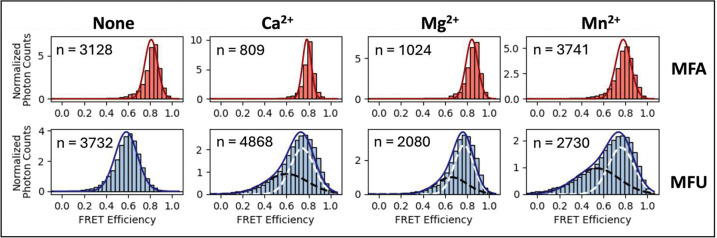
The FRET efficiency histograms are shown for both mid-FRET substrates MFA and MFU in each of the divalent ion conditions. The substrates were diluted to 5 nM in 30 mM HEPES, 100 mM NaCl, 5 mM DTT, and 5 mM of divalent cation chloride solution. The value of n indicated on each plot is the total number of bursts, or single molecule events, analyzed for that measurement. The FRET efficiency histograms for the MFA substrates were fit to single Gaussian distributions. All MFA FRET populations were narrow and centered at approximately E=0.8. The FRET efficiency histograms of MFU substrates have broader distributions than MFA and were fit to two-population Gaussian mixtures with peaks at E~0.6 and E~0.8. The Gaussian fit parameters are shown in Supplementary Table S11.

**Figure 5.**
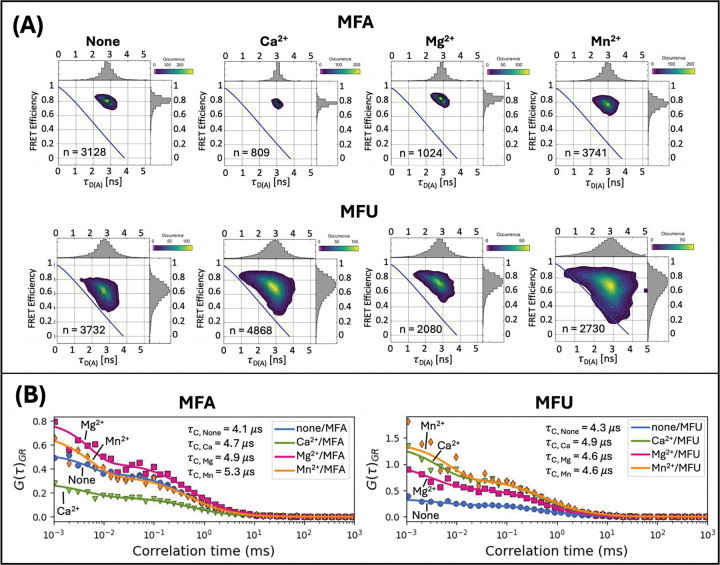
A) The FRET efficiency versus donor lifetime plots are shown for MFA and MFU without divalent cations and with 5 mM Ca^2+^, Mg^2+^, and Mn^2+^. The static FRET line in solid blue represents the theoretical location of static FRET states, Eq. 9. The MFA substrate population is well separated from the static FRET line under all conditions. The MFU substrate population has a broader FRET and lifetime distribution than any MFA population, particularly for Ca^2+^/MFU and Mn^2+^/MFU, and curves away from the static FRET line in all conditions. B) Autocorrelation curves for the isolated acceptor fluorescence signal after donor excitation (FRET, $I_{GR}$) are fit to a conformational model, Eq. 3, and the conformational time parameter ($t_{C}$) is shown for MFA and MFU incubated with no divalent ions and in 5 mM Ca^2+^, Mg^2+^ and Mn^2+^. The decay in the FCS data at microsecond timescales in all conditions is indicative of conformational dynamics of the substrates. The conformational times determined for MFA and MFU are similar.

Mid-FRET substrates (MFA and MFU) were designed to optimize the distance-dependent sensitivity of FRET efficiency (Figure 1B, Supplementary Table S1). The Förster distance of the AlexaFluor546-Atto647N FRET pair was estimated to be 64.13 Å from FPbase, and the separation between the donor and acceptor for MFA/MFU after annealing was approximately 60 Å. This corresponds to a minimum FRET efficiency of approximately 0.6. Each MFA or MFU sample was diluted to 5 nM in HEPES buffer containing the appropriate divalent cation and the PIE-FRET/FCS signal was collected for 60 seconds. We used a photon burst analysis algorithm in PAM [47] (Materials and Methods) to analyze single-molecule diffusion events. The resulting FRET efficiency histograms and 2-diminensional plots are shown in Figures 4 and 5. The value of n on each plot indicates the number of bursts analyzed, which represent single-molecule diffusion events. For all MFA experimental conditions, fitting the FRET efficiency distribution to a single Gaussian was sufficient, yielding E=0.8. (Figure 4, top). We expect that the MFA substrate would not be cleaved by Nsp15.

In contrast, introducing a single uridine in the MFU substrate produced a broader FRET efficiency distribution, which may indicate that the substrate samples multiple conformational states. Under all divalent cation conditions for the MFU substrate, each distribution fit to two Gaussians. The dominant FRET efficiency for none/MFU was E=0.6, which corresponds to the expected minimum FRET (Figure 4, bottom). The FRET efficiency distributions for MFU in the presence of Ca^2+^, Mg^2+^ and Mn^2+^ were broader with two peaks at E~0.6 and E~0.8 (Figure 4, bottom). The higher FRET efficiency states of Ca^2+^/MFU, Mg^2+^/MFU, and Mn^2+^/MFU compared to none/MFU suggest divalent ions induce a tighter conformation of the MFU substrate, whereas the apparent single E=0.8 FRET state of MFA appears unaffected by the change in divalent cation environment. The emergence of the high FRET state suggests a dynamic MFU structure that interconverts between the closed conformation (high FRET state) and the more open conformation (mid FRET state) [56] that dominates in the absence of divalent cations. The percentage of molecules in each conformational state can be estimated from the Gaussian fit parameters [68] (Supplementary Table S11). For MFU in the presence of Mn^2+^, the populations in the E=0.76 (closed) and E=0.54 (open) FRET states are nearly 50–50, compared to about 60–40 (closed to open) with Ca^2+^ and Mg^2+^. We propose that a slight perturbation to the nucleic acid energy landscape [69] may stabilize substrate conformations in MFU that promote cleavage by Nsp15 specifically at uridine depending on the local divalent cation environment.

Photon burst data can also be analyzed by calculating the donor fluorescence lifetime, which is quenched to varying degrees because of distance-dependent FRET to the acceptor molecule, Eq. 9. This time-resolved PIE-FRET analysis can be displayed with FRET efficiency versus donor lifetime (E vs. $t_{D(A)}$) histograms [57]. Figure 5A shows 2-dimensional plots of donor lifetime (tD(A)) versus FRET efficiency (E) for n number of bursts, or single-molecule events, for experimental conditions in the absence and presence of divalent cations. It is the same data analyzed for Figure 4. Deviation from the static FRET line (blue solid) is an indicator of dynamic behavior in the single molecules [57,70]. The static line is calculated using Eq. 9 and the measured values from Supplementary Table S2. MFA and MFU plots deviate from the static line, indicating that all the substrates are dynamic to some degree. The distributions for the MFU substrates are much broader, apparently sampling a larger range of stable conformational states, an effect that is most pronounced in the presence of manganese (Mn). For comparison, FCS and FRET Efficiency versus donor lifetime plots were generated for double-stranded benchmark DNA oligos [45] as a control for an expected static sample (Supplementary Figures S6, S7 and Table S12). In contrast to MFA/MFU, the E vs. $t_{D(A)}$ populations for benchmark DNA samples are on the static line (Figure S7). Notably, the benchmark DNA FCS curves (Figure S6) lack a microsecond timescale feature in the decay, thus are fit to a pure diffusion model. That is in stark contrast to HFA/HFU (Figure 2B) and MFA/MFU substrates (Figure 5B). Although only one FRET state could be resolved for MFA substrates (Figure 4, top), the deviation from the static line suggests this may be due to averaging of indistinguishable FRET states due to fast switching [41,57,71]. Next, we utilized FCS analysis to further characterize substrate dynamics and estimate conformational relaxation times.

We fit the autocorrelated FCS FRET signal ($I_{GR}$) for MFA and MFU substrates to a conformational model (Eq. 3) and extracted $t_{C}$, the conformational relaxation time (Figure 5B). The conformational relaxation times ($\tau_{C}$, Figure 5B) and additional conformational model fit parameters (Supplementary Table S13) were determined from the fitting. Autocorrelations of $I_{GG},\;I_{GR}$, and $I_{RR}$ and the corresponding fit parameters are in Supplementary Figure S8 and Table S14. The results in Figure 5B show that $t_{C}$ for MFA and MFU substrates are similar (~ 5 *m*s), indicating substrates with fast dynamic switching [48,61]. It is also interesting that the introduction of a single uridine leads to a broader FRET distribution. Taken together, these data suggest that uridine is structurally more flexible than adenine, and the role of manganese may be to induce substrate conformations that enable more efficient cleavage specifically at uridine. Divalent cations are known to interact with RNA by neutralizing the negative charge of the phosphate backbone, lowering the electrostatic repulsion between bases and increasing RNA flexibility [72,73]. This explains the emergence of higher FRET populations of MFU in the presence of divalent cations (Figure 4). The fast conversion time is consistent with the difficulty in resolving multiple FRET states due to the tendency of microsecond timescale dynamics to exhibit an averaged FRET state because of the rate of conversion [57,74,75]. Though dynamics appeared to be present in both MFA and MFU based on the E vs. $t_{D(A)}$ histograms (Figure 5A), the divalent cation environment affected MFU conformation more dramatically as evidenced by the shift in the FRET efficiencies for MFU in the presence of divalent cations, particularly in the case of Mn^2+^.

## DISCUSSION

**Figure 6.**
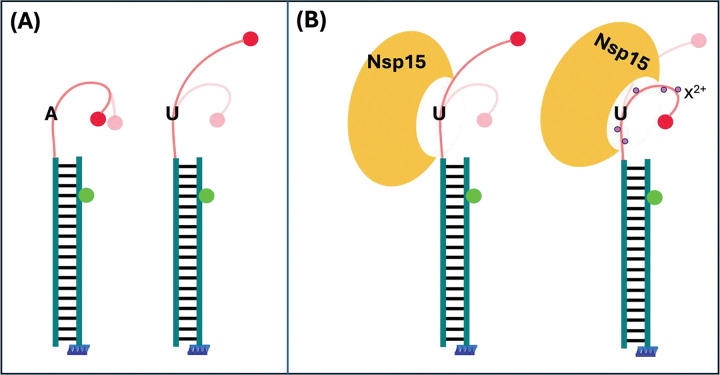
(A) Mid-FRET substrates MFA and MFU show different flexibility in general, where MFA (left) appears to adopt a more rigid closed conformation than substrate U as indicated by the higher FRET states of MFA in Figure 4. The average FRET of MFU in the absence of divalent cations is lower and broader, suggesting more flexibility is imparted by the uridine. (B) The conformational ensemble is composed of open (left) and closed (right) conformations of the substrate in the presence of divalent cations (purple). The open conformations are indicated by the low FRET population in Figure 4 (dashed black Gaussian curve), and higher FRET states of MFU are indicated by the dashed white Gaussian curves in Figure 4. The divalent cations (purple, $X^{2+}={Ca}^{2+},{Mg}^{2+}$, or $\left.{Mn}^{2+}\right)$ apparently induce a shift in the dynamic ensemble towards a population of the more closed conformation (right) that may allow the uridine in MFU to be more easily accessible within the 10-angstrom wide active site of Nsp15.

We have developed a combined PIE-FRET and FCS assay to explore the cleavage of RNA by Nsp15. Using our methodology and analysis strategies, we obtained clear indicators of cleavage by Nsp15 with both PIE-FRET and FCS data analysis. Our approach is also capable of differentiating between the cleavage activity of mutants and extracting kinetic rates. The ability to monitor the dynamic behavior of single molecules also revealed a structural impact on the RNA from the presence of uridine and divalent cations. PolyA and polyU tracts are known to have important biological functions, but the sequence and ion association dependence on structure is not well investigated experimentally. In a recent study, 30-base polyA and polyU ssRNA was investigated with UV-Vis, small angle X-ray scattering spectroscopy (SAXS), and circular dichroism (CD) in the presence of Na^+^ or Mg^2+^ [76]. The authors found that polyU was relatively unstructured under all conditions, while polyA was more compact and ordered due to base stacking. Notably, Mg^2+^ associated more strongly with polyA, producing a much larger effect on its conformation when compared to polyU. In other work, it has been shown that even single point modifications in nucleic acid sequences can impact backbone flexibility [77–79]. Thus, it is plausible that substituting a single A for U in our ssRNA substrates can increase ssRNA flexibility (Figure 6A), leading to the observed broad FRET efficiency histograms for MFU (Figure 4, 5A). Furthermore, divalent cations may associate less with the single uridine in HFU and MFU substrates, leading to a less compact and more dynamic structure surrounding the cleavage site. This is consistent with the breadth in FRET efficiency histograms we observed for MFU in the presence of all divalent cations (but not MFA). Differences in the conformational dynamics and ion atmosphere may make U more accessible for Nsp15 binding and cleavage [76,78,80]. For the more dynamic MFU substrate, we observe one population in the FRET efficiency distribution (E~0.6) without divalent cations and two populations (E~0.6 and 0.8) in the presence of divalent cations (Figure 4, Figure 6B). We propose that the E~0.6 population is more open and dynamic, but the E~0.8 population is compact due to the reduced backbone repulsion and thus its dynamics are constrained. The local area around the U may remain slightly flexible in the presence of divalent cations, which would allow Nsp15 to capture the U in its 10-Angstrom active site as a flipped base or bulge.

Over the past several years, the specificity of Nsp15 has been investigated through biochemical, structural, and high-throughput sequencing approaches [11,15,17–19,23,81]. Collectively, this work supports that Nsp15 is active across a broad range of ss- and dsRNA substrates. Our work establishes that the dynamic behavior of the substrate likely represents a new important variable for specificity. This agrees with recent studies showing that Nsp15 preferentially cleaves RNA in less thermodynamically stable areas [18,23]. RNA molecules readily fold into complex secondary and tertiary structures that exist as an ensemble of many conformations in solution [27,77,82,69,83]. The complex hierarchical free energy landscape leads to structural dynamics across 12 orders of magnitude from picoseconds to seconds. The presence of metabolites, ions, proteins, and other environmental conditions can perturb the energy landscape and shift the conformational population, and thus structural dynamics, from one that favors protection to one that favors cleavage.

In conclusion, we developed biophysical approaches to investigate the nuclease activity of Nsp15. Our method requires nanomolar or less concentrations of nucleic acid and protein. Future applications of these approaches include investigating the impact of inhibitors on Nsp15 activity and investigating the structural dynamics of highly conserved RNA sequences found in coronaviruses [84] for therapeutic applications. More broadly, our approach can be used to explore the impact of inhibitors that target RNA on its dynamics [85]. From our data analysis, the Nsp15-RNA interaction during cleavage appears to be very transient. A more complete understanding of the Nsp15 cleavage mechanism can be gleaned from fluorescently labeling and monitoring the enzyme itself. Nsp15 is homo-hexameric, thus site-specific labeling for FRET will require advanced strategies. Diffusion-based single-molecule fluorescence measurements present exciting opportunities to explore fast dynamics across many timescales that are relevant for understanding biomolecular interactions.

## Supplementary Material

SUPPLEMENTARY DATA

Supplementary data are available online.

Supplementary Files

This is a list of supplementary files associated with this preprint. Click to download.

• GordonetalNsp15SRPSupplementalData09252025clean.pdf

• 20210816cov3cov30prepgel3.png

## Figures and Tables

**Figure 1.**
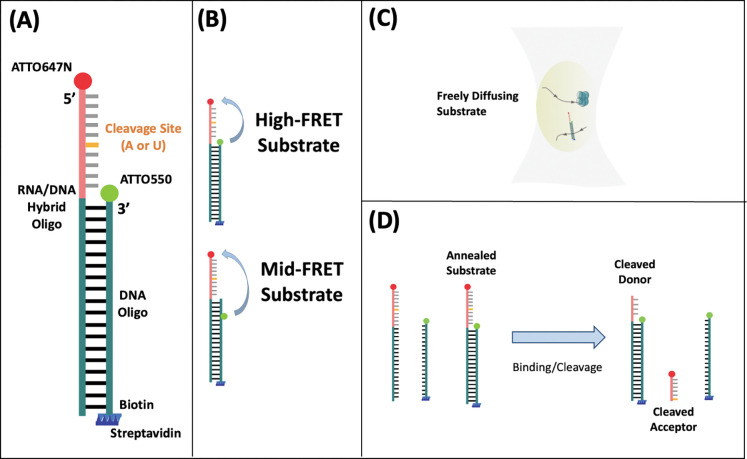
(A) Cleavage substrates are composed of an RNA(pink)/DNA(green) hybrid oligo and a complementary DNA strand (green). The complementary DNA strand is labeled with a FRET donor (either Atto550 or AlexaFluor546 – AF546), and the RNA segment of the hybrid is labeled with a FRET acceptor (Atto647N). A cleavage site (either an A or U nucleotide) is located within the RNA strand. The DNA acceptor strand is biotinylated at the 5’ end for attachment to streptavidin for future immobilized substrate assays. (B) Annealed double-labeled substrates are either high FRET (HFA/HFU), in which the complementary DNA strand is end labeled with Atto550, or mid-FRET (MFA/MFU), in which the complementary DNA strand is labeled in the backbone with AF546. (C) Intensity data is collected from individual substrates that freely diffuse in solution and traverse the observation volume. (D) Several subpopulations may be present in solution over the course of the 2-hour reaction. Prior to cleavage by Nsp15, annealed substrate samples may contain small amounts of unannealed oligos due to imperfect annealing efficiency. After cleavage, acceptor-labeled fragments and larger cleaved donor fragments remain in solution.

**Figure 1.**
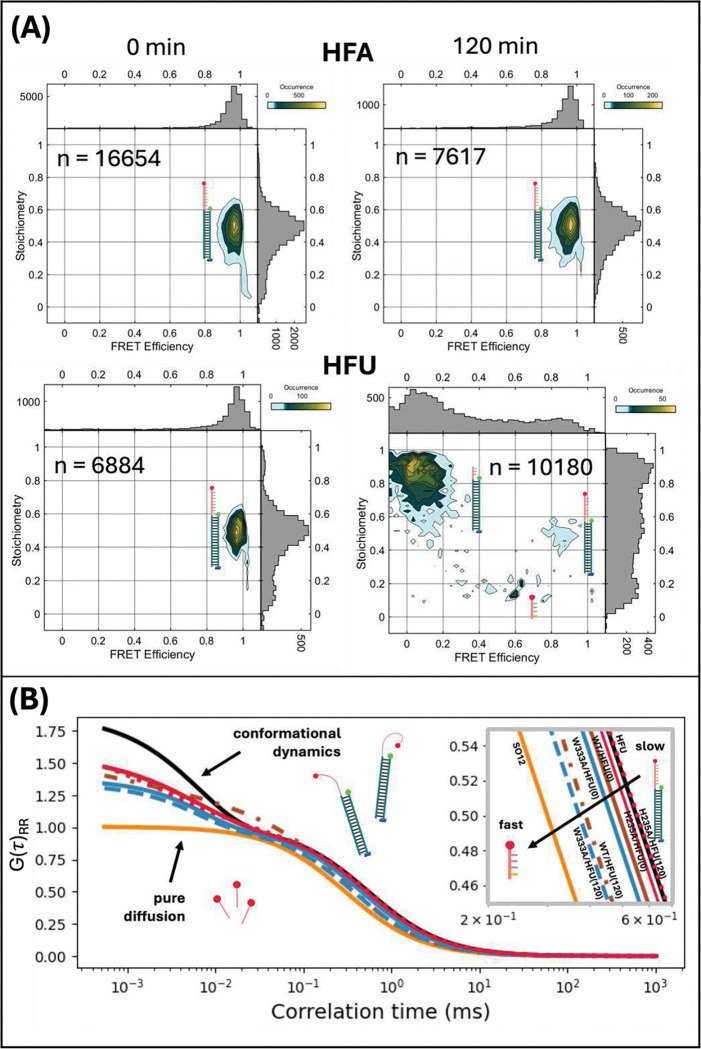
(A) Left: Two-dimensional FRET efficiency (E) vs. photon stoichiometry (S) histograms of annealed high-FRET substrates (HFA and HFU) prior to cleavage by wildtype Nsp15, where populations at E~1 and S=0.5 are consistent with intact substrates. After 2 hours of incubation with WT Nsp15, the high FRET efficiency population remained for the HFA substrate, but donor-only (S>0.8) and acceptor-only (S<0.2) populations appeared for the HFU substrate, consistent with HFU substrate cleavage by Nsp15. (B) FCS fits of I_RR_ signal autocorrelations for HFU incubated without or with Nsp15 variants. An FCS fit of the HFU substrate observed without the addition of Nsp15 (black) exhibits a pronounced decay near the microsecond timescale, likely consistent with conformational changes in the substrate. The FCS fit of the HFU substrate incubated with WT Nsp15 (solid brown, hidden by the solid red curve, visible inset and in Supplementary Figure S4) demonstrates a decay feature with a microsecond timescale upon initial introduction of WT Nsp15 and the microsecond decay feature is reduced after 2 hours (dashed brown). The FCS fit of HFU incubated with WT Nsp15 after 2 hours (dashed brown) is similar to that of the 12-base RNA oligo (SO12, orange) that we measured as a control for the completely cleaved RNA fragment, suggesting that near-complete substrate cleavage has occurred. FCS fits of the HFU substrate incubated with W333A Nsp15 (solid blue, dashed blue) suggest less efficient cleavage. HFU incubated with H235A (solid red, dashed red) demonstrate conformational changes of the substrate initially and after a 2-hour incubation with Nsp15, suggesting that cleavage did not occur. Inset: The millisecond-timescale diffusion regime of the FCS fits shows slower translational diffusion (right shifted decays) for intact substrates, and faster diffusion (left shifted decays) for cleaved substrates. The FCS curves are normalized to $G(2\cdot \tau_{D})$. Raw correlation curves with fits for 2B are shown in Supplementary Figure S4, and fit parameters $1/N,\;\tau_{D},\;A_{C},\;\tau_{C}$ and are included in Supplementary Table S5.

## Data Availability

Raw datasets (.ptu files) are available in Dryad data repository at DOI: 10.5061/dryad.573n5tbjz. A README file is included that explains experimental conditions and acquisition parameters. Open-source PIE Analysis with MATLAB (PAM; The MathWorks) software (https://pam.readthedocs.io/en/latest/GettingStarted.html) can open and process .ptu files. PAM software can be downloaded from GitLab (https://gitlab.com/PAM-PIE/PAMcompiled). Python code is available upon request.

## STATEMENT OF SIGNIFICANCE

Nsp15, a protein conserved among coronaviruses, cleaves uridine-rich viral RNA sequences to mitigate the host cell immune response. This function makes Nsp15 an ideal target for broad-acting therapeutics for coronavirus diseases. We establish diffusion-based single-molecule PIE-FRET and FCS methods to monitor substrate cleavage and dynamics simultaneously. To the best of our knowledge, this is the first assay developed that can monitor both aspects of the Nsp15-substrate interaction. Analysis of time-resolved data reveals that substitution of a single uridine in place of an adenine imparts flexibility into the substrate, an effect that is exacerbated with divalent cations. Our findings support previous work on Nsp15 specificity but adds a new puzzle piece that substrate flexibility is an important consideration for nuclease activity and specificity.
